# Association between the Infant and Child Feeding Index (ICFI) and nutritional status of 6- to 35-month-old children in rural western China

**DOI:** 10.1371/journal.pone.0171984

**Published:** 2017-02-16

**Authors:** Pengfei Qu, Baibing Mi, Duolao Wang, Ruo Zhang, Jiaomei Yang, Danmeng Liu, Shaonong Dang, Hong Yan

**Affiliations:** 1 Xi’an JiaoTong University, Health Science Center, Xi’an, Shaanxi Province, P.R. China; 2 Department of Clinical Sciences, Liverpool School of Tropical Medicine Pembroke Place, Liverpool, United Kingdom; 3 Nutrition and Food Safety Engineering Research Center of Shaanxi Province, Xi'an, Shaanxi Province, P.R. China; Universidade de Sao Paulo, BRAZIL

## Abstract

**Background:**

The objective of this study was to determine the relationship between the quality of feeding practices and children’s nutritional status in rural western China.

**Methods:**

A sample of 12,146 pairs of 6- to 35-month-old children and their mothers were recruited using stratified multistage cluster random sampling in rural western China. Quantile regression was used to analyze the relationship between the Infant and Child Feeding Index (ICFI) and children’s nutritional status.

**Results:**

In rural western China, 24.37% of all infants and young children suffer from malnutrition. Of this total, 19.57%, 8.74% and 4.63% of infants and children are classified as stunting, underweight and wasting, respectively. After adjusting for covariates, the quantile regression results suggested that qualified ICFI (ICFI > 13.8) was associated with all length and HAZ quantiles (P<0.05) and had a greater effect on the following: poor length and HAZ, the β-estimates (length) from 0.76 cm (95% CI: 0.53 to 0.99 cm) to 0.34 cm (95% CI: 0.09 to 0.59 cm) and the β-estimates (HAZ) from 0.17 (95% CI: 0.10 to 0.24) to 0.11 (95% CI: 0.04 to 0.19). Qualified ICFI was also associated with most weight quantiles (P<0.05 except the 80th and 90th quantiles) and poor and intermediate WAZ quantiles (P<0.05 including the 10th, 20th 30th and 40th quantiles). Additionally, qualified ICFI had a greater effect on poor weight and WAZ quantiles in which the β-estimates (weight) were from 0.20 kg (95% CI: 0.14 to 0.26 kg) to 0.06 kg (95% CI: 0.00 to 0.12 kg) and the β-estimates (WAZ) were from 0.14 (95% CI: 0.08 to 0.21) to 0.05 (95% CI: 0.01 to 0.10).

**Conclusions:**

Feeding practices were associated with the physical development of infants and young children, and proper feeding practices had a greater effect on poor physical development in infants and young children. For mothers in rural western China, proper guidelines and messaging on complementary feeding practices are necessary.

## Introduction

Malnutrition, a prevalent disease in children in developing counties, seriously affects children's growth, development and health. Improving children's nutritional status and reducing the prevalence of malnutrition to promote their physical and mental development are greatly important [1,2]. In the 2016 State of the World's Children report, the United Nations Children's Fund (UNICEF) noted that the prevalence of moderate or severe stunting in children under 5 years old was 9%, and the prevalence of moderate or severe underweight was 3% in China [3]. China's western region is sparsely populated with complex terrain, economic stagnation and low levels of maternal and child health care. Therefore, understanding the nutritional status of infants and children in western China and exploring the factors that influence it are important for improving infant and child nutrition and growth in this region. From 2001 to 2005, the Chinese Ministry of Health (MOH) and UNICEF performed a Rural Primary Health Care Project (PRHC) in forty-five counties of 10 rural, western Chinese provinces. Using program data from 2005, the objective of this study was to analyze the relationship between the Infant and Child Feeding Index (ICFI) and children’s nutritional status in rural western China in 2005.

## Materials and methods

### Study design and participants

This cross-sectional study, part of the Rural Primary Health Care Project, was conducted in rural western China in 2005. In total, 14,112 women and children from ten provinces were enrolled, and a written informed consent was obtained from each adult participant at the start of the survey. Considering the hierarchical structure of Chinese administrative districts and the varied population distributions among the provinces, a stratified 3-stage probability proportional to size sampling method was employed in this study. Forty-five counties were selected in terms of social and economic development by MOH and UNICEF. All counties were poor economy and higher willing-ness to participate in the project. Within each county, five townships were randomly selected; within each township, four villages were randomly selected. Within each village, 16 families were randomly selected, from which a woman with children under the age of 3 was selected to participate in the study. All of the women were interviewed in-person by trained professional interviewers from the Xi’an Jiaotong University College of Medicine.

The World Health Organization (WHO) recommends that babies receive nutritionally adequate and safe complementary foods after 6 months of exclusive breastfeeding. There were less than 60 children over 3 years old in this study. Taking into account the WHO recommendations about complementary feeding and this composition of children’s ages, mothers and children below 6 months old and aged 36 months or older were eliminated from the study. The inclusion criteria for participation in the study was that child were 6–35 months old and mother consented to participate in this project. And exclusion criteria were that child was less than 6 months old or child was aged 36 months or older and mother refused to participate in this project. The study included 12,146 pairs of mothers and their children between the ages of 6 and 35 months.

### Assessment of child nutritional status

Anthropometric measurements, including height and weight, were used to assess infant and child nutrition. The recumbent lengths of all infants and children in the study were measured with a standard calibrated board accurate to the nearest 1 mm (Model WB-II, Beijing Tractor Company No. 6 Measuring Factory, Beijing, China). Tared weighting was used to measure child's weight. Mother was to be weighted first alone, and mother with handing child was to be weight secondly. The difference of twice weight was child’s weight. All children were weighted with minimal clothing. All weights were measured to the nearest 500 g with a standard calibrated balance scale (Model YGZ-12, Wuxi Measurement Factory, Wuxi, China). Measurements were performed according to recommended standard methods by trained interviewers. Nutritional status was assessed based on the WHO Multicenter Growth Reference Study Standards (2006). We calculated the Z-scores of height for age (HAZ), weight for age (WAZ) and weight for height (WHZ) based on the WHO guidelines. Malnutrition was assessed based on the WHO Child Growth Standards (WHO, 2006) [4].

stunting–proportion of children with height-for-age below –2 standard deviations (SD);
underweight–proportion of children with weight-for-age below –2 SD;
wasting–proportion of children with weight-for-height below –2 SD;
Malnutrition in children was defined as stunting, underweight, or wasting.

### Feeding information and definition

All of the participants were interviewed in-person, and data were collected on the children’s health care, morbidity, anthropometry, and feeding practices, as well as the mother’s health care. Moreover, data were also collected on related information such as family background and living environment. When necessary, fathers were asked to help the mothers provide family background information. A specially designed family questionnaire was used for the data collection. Family questionnaire covered breastfeeding practice including initial time and duration of breastfeeding and complementary feeding practice including time, frequency and type of introduced food. According to WHO complementary feeding guiding principle of infant and young children and related study of infant and young children feeding in Western China, this study used introduced time and frequency of grains, egg, fresh milk, formula, bean products, fish, meat (beef, mutton and pork), fruits and vegetables to represent complementary feeding practice [5–7]. In this study, we mainly utilized the feeding data, which included information on complementary feeding practices. All of the feeding information was obtained through interviews with the mothers.

The following definitions of nutrition and feeding indicators are used in this paper:

‘Infant and Child Feeding Index’: Based on initial findings, the ICFI was developed using the following: a method proposed by Ruel and Menon [8], an ICFI scoring system developed by Lai Jian-qiang and the Nutrition and Health Survey of the Chinese People [9], and the WHO feeding recommendations. The ICFI was separated by the 3 age groups: 6–8.99 months, 9–11.99 months and 12–35.99 months (Table 1). To take into account the recommendations of the National Institute for Nutrition and Health, Chinese Center for Disease Control and Prevention, qualified ICFI refers to a score exceeding 60% of the total ICFI score. In this paper, qualified ICFI > 13.8 and unqualified ICFI ≤ 13.8 (Table 1).

**Table 1:** Components and scores of ICFI by age group.

Variables	6~8 months	9~11 months	12~35 months
Breastfeeding	No = 0	No = 0	No = 0
	Yes = 2	Yes = 2	Yes = 1^a^
Bottle feeding	No = 1	No = 1	No = 1
	Yes = 0	Yes = 0	Yes = 0
Grains	≤1 time/month = 0	≤1 time/month = 0	≤1 time/month = 0
	2–3 times/month~1 time/week = 1	2–3 times/month~1 time/week = 1	2–3 times/month~1 time/week = 1
	≥2–3 times /week = 2	≥2–3 times /week = 2	≥2–3 times /week = 2
Egg	≤1 time/month = 0	≤1 time/month = 0	≤1 time/month = 0
	2–3 times/month~1 time/week = 1	2–3 times/month~1 time/week = 1	2–3 times/month~1 time/week = 1
	≥2–3 times /week = 2	≥2–3 times /week = 2	≥2–3 times /week = 2
Milk	≤1 time/month = 0	≤1 time/month = 0	≤1 time/month = 0
	2–3 times/month~1 time/week = 1	2–3 times/month~1 time/week = 1	2–3 times/month~1 time/week = 1
	≥2–3 times /week = 2	≥2–3 times /week = 2	≥2–3 times /week = 2
			≥4 times /week = 3
Formula	≤1 time/month = 0	≤1 time/month = 0	≤1 time/month = 0
	2–3 times/month~1 time/week = 1	2–3 times/month~1 time/week = 1	2–3 times/month~1 time/week = 1
	≥2–3 times /week = 2	≥2–3 times /week = 2	≥2–3 times /week = 2
Bean products	≤1 time/month = 0	≤1 time/month = 0	≤1 time/month = 0
	2–3 times/month~1 time/week = 1	2–3 times/month~1 time/week = 1	2–3 times/month~1 time/week = 1
	≥2–3 times /week = 2	≥2–3 times /week = 2	≥2–3 times /week = 2
Fish	≤1 time/month = 0	≤1 time/month = 0	≤1 time/month = 0
	2–3 times/month~1 time/week = 1	2–3 times/month~1 time/week = 1	2–3 times/month~1 time/week = 1
	≥2–3 times /week = 2	≥2–3 times /week = 2	≥2–3 times /week = 2
Meat	≤1 time/month = 0	≤1 time/month = 0	≤1 time/month = 0
(beef, mutton and pork)	2–3 times/month~1 time/week = 1	2–3 times/month~1 time/week = 1	2–3 times/month~1 time/week = 1
	≥2–3 times /week = 2	≥2–3 times /week = 2	≥2–3 times /week = 2
Fruits and vegetables	≤1 time/month = 0	≤1 time/month = 0	≤1 time/month = 0
	2–3 times/month~1 time/week = 1	2–3 times/month~1 time/week = 1	2–3 times/month~1 time/week = 1
	≥2–3 times /week = 2	≥2–3 times /week = 2	≥2–3 times /week = 2
First complementary feeding time	<4 months or ≥9 months = 0	<4 months or ≥9 months = 0	<4 months or ≥9 months = 0
	4~5 months = 1	4~5 months = 1	4~5 months = 1
	6~8 months = 2	6~8 months = 2	6~8 months = 2
Dietary diversity	0 kind = 0	0 kind = 0	0 kind = 0
(egg, milk, bean products, fish, meat, and others ^b^)	1~3 types = 1	1~3 types = 1	1~3 types = 1
	≥4 types = 2	≥4 types = 2	≥4 types = 2
Total scores	23	23	23
Qualified ICFI	> 13.8	> 13.8	> 13.8
Unqualified ICFI	≤13.8	≤13.8	≤13.8

a: assign ‘Breastfeeding Yes = 1’ because the infant at 12–35 months had difficulty continuing breastfeeding.

b: ‘others’ includes grains, formula, fruits and vegetables.

### Quality control

All of the interviewers were trained with the same guidelines before the study. A quality control system was constructed during the study, and interviewers checked themselves and were checked by other interviewers and by a supervisor. Re-interviews were necessary when logistical questions or missing values were found.

### Statistical analysis

The sample size was 12,146 pairs of mothers and their children. A database was designed using EpiData Version 3.02 (The EpiData Association, Odense, Denmark), and data entries were duplicated. To identify the economic status of the participants, a wealth index was established using a principal component analysis of household assets, facilities and income (Household Vehicles: none, bicycle, motorcycle, tractor or car; Household Television Sets: none, black and white television sets or color television sets; Water Source: tap water or not; Household Income: farming or animal husbandry only or farming or animal husbandry and others). This procedure standardized the indicator variables (calculating z-scores); the factor coefficient scores (factor loadings) were calculated; and, for each household, the indicator values were multiplied by the loadings and summed to produce the household’s index value. In this process, the first of the factors produced was used to represent the wealth index, and participants’ economic status was categorized into 3 groups: poor, moderate, and rich [10]. Initially, the descriptive data on participants’ characteristics were summarized using the mean and standard deviation for continuous variables. Counts and proportions were used for the categorical variables. Chi-squared tests were performed to compare the categorical variables. To compare continuous variables among the groups, an analysis of covariance (normally distributed variables) or the Kruskal-Wallis test (abnormally distributed variables) were used.

Generalized estimating equations (GEE) model was used to analyze the relationship between ICFI and children’s nutritional status. Ethnic group, province, county rank, wealth index, mother’s education, mother’s age, parity, child's gender and child's age were variables that were controlled for in GEE models.

Quantile regression (QR) extends a regression model to conditional quantiles of the response variable, such as the 90th percentile. Quantile regression is particularly useful when the rate of change in the conditional quantile, expressed by the regression coefficients, depends on the quantile. An advantage of quantile regression over a least squares regression is its flexibility in modeling data that have heterogeneous conditional distributions [11–14]. Quantile regression was used to detect an association between qualified ICFI and children’s nutritional status (length, weight, HAZ, WAZ and HAZ). In the quantile regression, 9 quantiles of length, weight, HAZ, WAZ and WHZ were selected (10th to 90th, step by 10th) from lowest to highest children’s nutritional status. Regression coefficients for each quantile and their 95% confidence intervals (95% CI) were computed for qualified ICFI using unqualified ICFI as a reference. The multivariate linear regression, which was based on the ordinary least squares (OLS), explained the mean value of children’s nutritional change by qualified ICFI. Ethnic group, province, county rank, wealth index, mother’s education, mother’s age, parity, child's gender and child's age were adjusted in the quantile regression.

All of the analyses were performed with SPSS Version 13.0 (Statistical Package For Social Science, Inc., Chicago, IL, USA) and SAS 9.3 (SAS Institute Inc., Cary, NC). The level of significance was set at *p*<0.05.

### Ethics statement

The ethics committee of Xi’an Jiaotong University College of Medicine approved this study. Written informed consent was obtained from all of the adult participants in this study after a detailed explanation of the research.

## Results

### Sociodemographic characteristics and nutritional status of children

The details of the sample and distribution of the major demographic variables are shown in Table 2. There were 7,800 Han; 1,060 Uygur; 712 Tibetan; 610 Zhuang; and 1964 other minority children. The greatest number of children came from Xinjiang province, while the least number of children came from Gansu province. There were 3,175 children aged 6–11 months; 5,151 children aged 12–23 months; and 3,820 children aged 24–35 months. There was no clustering in children’s age.

**Table 2:** Sociodemographic characteristics and nutritional status^a^ of children in rural western China, 2005.

		N	%	Length (cm)	Weight (kg)	HAZ (z-score)	WAZ (z-score)	WHZ (z-score)	Stunting (%)	Underweight (%)	Wasting (%)	Malnutrition (%)
Ethnic groups												
	Han	7,800	64.22	79.76±8.13	10.39±1.99	-0.73±1.41	-0.40±1.13	0.02±1.16	16.24	6.77	4.18	20.69
	Uygur	1,060	8.73	78.15±7.36	9.82±1.91	-1.16±1.36	-0.82±1.06	-0.28±1.00	23.48	11.80	5.42	28.49
	Tibetan	712	5.86	78.94±7.68	10.54±1.95	-1.18±1.59	-0.37±1.13	0.39±1.20	28.92	7.17	2.55	32.01
	Zhuang	610	5.02	76.04±7.14	9.28±1.77	-1.30±1.32	-1.00±1.13	-0.40±1.26	29.42	17.08	8.11	37.23
	Others	1,964	16.17	78.84±7.91	10.16±2.08	-1.01±1.46	-0.59±1.26	-0.07±1.27	23.55	12.44	5.55	29.20
	P value			<0.001	<0.001	<0.001	<0.001	<0.001	<0.001	<0.001	<0.001	<0.001
Province												
	Gansu	575	4.73	81.21±8.90	10.82±1.98	-0.96±1.68	-0.41±1.06	0.08±1.16	23.30	6.71	3.99	26.79
	Guangxi	1,296	10.67	77.18±7.34	9.66±1.84	-1.05±1.30	-0.73±1.16	-0.24±1.25	21.40	12.53	6.85	27.97
	Guizhou	1,119	9.21	78.21±7.53	9.68±1.95	-1.43±1.49	-1.12±1.15	-0.49±1.17	35.55	21.45	8.86	43.91
	Jiangxi	1,289	10.61	78.80±7.54	10.16±1.94	-0.74±1.35	-0.46±1.20	-0.09±1.26	15.81	8.88	6.12	22.58
	Inner Mongolia	1,021	8.41	79.93±8.75	10.57±2.05	-0.24±1.23	-0.02±1.03	0.16±1.01	6.72	2.16	1.28	8.58
	Ningxia	1,112	9.16	80.88±8.12	10.59±1.99	-0.36±1.35	-0.25±1.12	-0.05±1.15	7.45	4.23	3.09	11.33
	Qinghai	1,427	11.75	79.49±7.78	10.40±1.91	-0.81±1.35	-0.36±1.04	0.10±1.09	17.90	4.85	2.61	20.94
	Sichuan	1,374	11.31	79.48±7.96	10.33±2.02	-1.07±1.60	-0.59±1.25	-0.02±1.36	26.71	10.88	6.57	33.46
	Xinjiang	1,827	15.04	79.28±8.20	10.41±2.15	-0.89±1.40	-0.40±1.16	0.10±1.15	18.71	7.64	3.66	22.28
	Chongqing	1,106	9.11	79.57±8.00	10.31±1.89	-1.15±1.32	-0.62±0.99	-0.01±1.01	24.14	6.28	2.31	26.52
	P value			<0.001	<0.001	<0.001	<0.001	<0.001	<0.001	<0.001	<0.001	<0.001
County rank												
	Ⅰ	2,378	19.58	80.14±8.52	10.64±2.13	-0.55±1.31	-0.18±1.12	0.15±1.20	12.24	5.17	3.64	16.27
	Ⅱ	2,401	19.77	79.35±7.68	10.28±1.90	-0.84±1.44	-0.49±1.16	-0.04±1.22	18.06	8.08	4.68	22.83
	Ⅲ	5,465	44.99	79.12±8.05	10.22±1.99	-0.91±1.45	-0.52±1.16	-0.05±1.18	20.74	8.74	4.56	25.48
	Ⅳ	1,902	15.66	78.17±7.46	9.82±1.91	-1.21±1.45	-0.85±1.12	-0.27±1.10	27.53	14.01	5.94	33.46
	P value			<0.001	<0.001	<0.001	<0.001	<0.001	<0.001	<0.001	0.007	<0.001
Wealth index												
	Poor	1,489	12.26	78.51±7.70	9.92±1.97	-1.32±1.47	-0.91±1.21	-0.24±1.17	32.09	16.94	7.07	38.81
	Moderate	3,283	27.03	78.98±7.88	10.16±2.02	-1.10±1.46	-0.67±1.16	-0.10±1.23	25.23	10.87	5.47	30.82
	Rich	7,374	60.71	79.47±8.10	10.36±2.00	-0.68±1.38	-0.34±1.12	0.01±1.17	14.55	6.15	3.77	18.60
	P value			<0.001	<0.001	<0.001	<0.001	<0.001	<0.001	<0.001	<0.001	<0.001
Parity												
	1	7,083	58.32	79.20±8.15	10.25±2.02	-0.80±1.40	-0.46±1.15	-0.03±1.17	17.84	7.95	4.19	22.38
	2	4,337	35.71	79.22±7.78	10.26±1.97	-0.94±1.47	-0.55±1.18	-0.06±1.21	21.35	9.66	5.19	26.65
	> = 3	726	5.98	79.42±7.88	10.31±2.10	-1.11±1.44	-0.64±1.16	-0.10±1.19	26.07	10.93	5.57	30.44
	P value			0.791	0.723	<0.001	<0.001	0.291	<0.001	0.001	0.028	<0.001
Child's gender												
	Male	7,011	57.72	79.80±7.88	10.50±2.00	-0.92±1.43	-0.53±1.19	-0.05±1.22	21.05	9.36	5.19	26.28
	Female	5,135	42.28	78.42±8.10	9.91±1.97	-0.80±1.43	-0.46±1.13	-0.04±1.14	17.57	7.89	3.87	21.78
	P value			<0.001	<0.001	<0.001	0.003	0.662	<0.001	0.005	<0.001	<0.001
Child's age (month)												
	6–11	3,175	26.14	70.37±4.27	8.47±1.32	-0.30±1.47	-0.17±1.28	0.05±1.30	9.75	6.68	5.31	15.83
	12–23	5,151	42.41	78.82±4.91	10.06±1.49	-0.87±1.41	-0.53±1.14	-0.13±1.18	19.03	8.77	5.18	24.00
	24–35	3,820	31.45	87.28±5.08	12.03±1.59	-1.35±1.25	-0.75±1.02	-0.02±1.07	28.63	10.44	3.30	32.08
	P value			<0.001	<0.001	<0.001	<0.001	<0.001	<0.001	<0.001	<0.001	<0.001
Mother's education												
	No education	4,278	35.22	78.87±7.77	10.14±1.98	-1.09±1.45	-0.65±1.19	-0.09±1.19	24.67	11.53	5.27	29.77
	Primary school	4,358	35.88	79.26±8.07	10.29±2.03	-0.84±1.45	-0.47±1.16	-0.02±1.21	19.19	8.22	4.63	23.97
	Junior high school	2,918	24.02	79.43±8.11	10.28±1.97	-0.70±1.33	-0.41±1.10	-0.05±1.14	14.61	6.40	4.04	19.37
	Senior high school and above	592	4.87	80.36±8.51	10.64±2.13	-0.38±1.39	-0.13±1.14	0.12±1.17	10.42	4.15	2.95	13.37
	P value			0.001	<0.001	<0.001	<0.001	<0.001	<0.001	<0.001	0.023	<0.001
Mother's age (year)												
	<25	4,293	35.34	77.78±7.85	9.93±1.94	-0.80±1.40	-0.47±1.14	-0.04±1.17	18.08	7.88	4.06	22.74
	25–29	4,564	37.58	80.15±7.87	10.48±2.01	-0.90±1.46	-0.50±1.18	-0.03±1.19	20.05	9.14	4.84	24.97
	> = 30	3,289	27.08	79.82±8.13	10.37±2.04	-0.92±1.44	-0.53±1.16	-0.07±1.21	20.90	9.31	5.09	25.69
	P value			<0.001	<0.001	<0.001	0.087	0.389	0.007	0.051	0.084	0.008
ICFI												
	Qualified	6,004	49.43	80.43±7.66	10.51±1.94	-0.82±1.40	-0.46±1.11	-0.04±1.16	17.68	7.43	4.12	22.01
	Unqualified	6,142	50.57	77.94±8.15	9.98±2.04	-0.92±1.47	-0.54±1.22	-0.05±1.22	21.60	10.13	5.18	26.90
	P value			<0.001	<0.001	<0.001	<0.001	0.541	<0.001	<0.001	0.007	<0.001
	OR^a^	-	-	-	-	-	-	-	0.77	0.75	0.81	0.76
	95% CI	-	-	-	-	-	-	-	0.69–0.85	0.64–0.86	0.67–0.98	0.69–0.84
TOTAL		12,146	100.00	79.22±8.00	10.25±2.01	-0.87±1.43	-0.50±1.16	-0.05±1.19	19.57	8.74	4.63	24.37

a: ref = Unqualified ICFI; ethnic group, province, county rank, wealth index, mother’s education, mother’s age, parity, child's gender and child's age were adjusted in GEE models.

**Fig 1:**
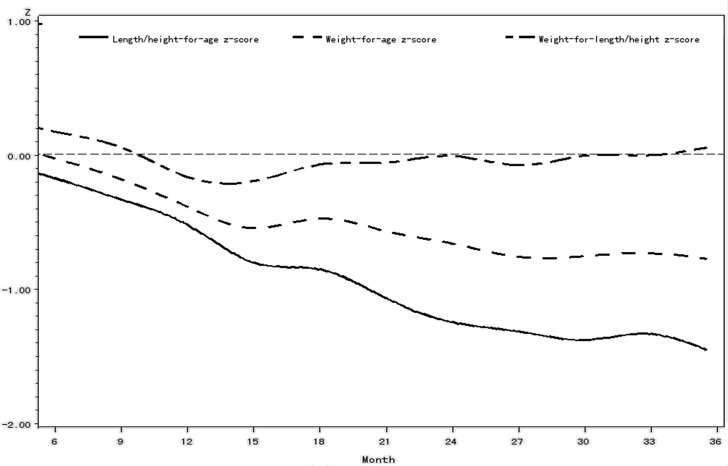
Comparison of HAZ, WAZ, and WHZ trends in different age groups of children in rural western China, 2005.

In rural western China, 24.37% of infants and young children had malnutrition. Stunting was found in 19.57%; underweight was found in 8.74%; and wasting was found in 4.63% of infants and children. Significant differences were found in children’s length, weight, HAZ, WAZ, WHZ, stunting rate, underweight rate, wasting rate and malnutrition rate among different ethnic groups, provinces, county rank groups, wealth index groups, children’s age groups (Fig 1), and mothers’ educational levels (P<0.05). There were no differences in children’s length, weight and WHZ among different parity groups (P>0.05). There were no differences in children’s WHZ by gender (P>0.05). There were no differences in children’s WAZ, WHZ, underweight rate and wasting rate among mothers’ age groups(P>0.05).

**Fig 2:**
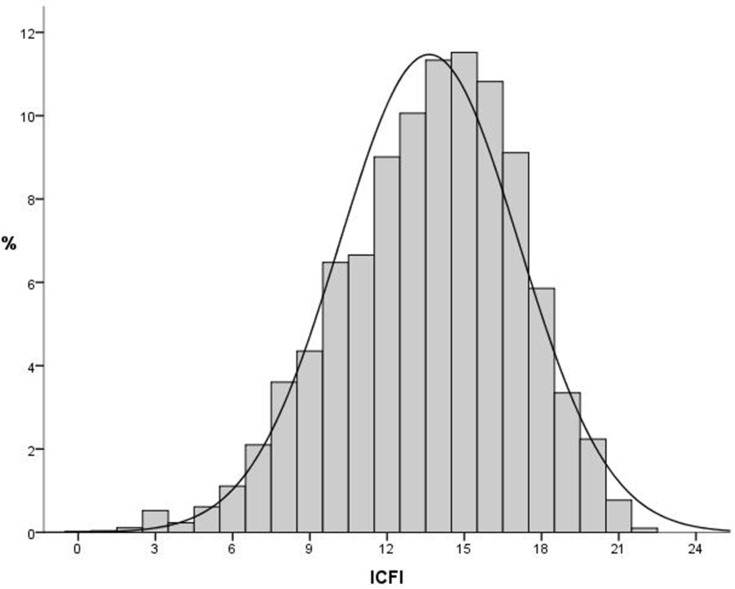
Distribution of ICFI of children in rural western China, 2005.

The distribution of ICFI of children in the study was similar to a normal distribution (Fig 2). The mean of the ICFI was 13.64, and the standard deviation was 3.48. The median of the ICFI was 14. The top three ICFI scores were 14, 15, and 16, accounting for 10.17%, 10.33% and 9.71% of the total, respectively.

### Association between qualified ICFI and children’s malnutrition

In Table 2, generalized estimating equations models indicated that qualified ICFI were protective factors for child malnutrition. Generalized estimating equations models showed that children with qualified ICFI were less likely to be classified as stunting, underweight, wasting and overall malnutrition than children with unqualified ICFI (OR = 0.77, 95% CI = 0.69–0.85; OR = 0.75, 95% CI = 0.64–0.86; OR = 0.81, 95% CI = 0.67–0.98; OR = 0.76, 95% CI = 0.69–0.84). Related variables (ethnic group, province, county rank, wealth index, mother’s education, mother’s age, parity, child's gender and child's age) were adjusted in GEE models.

### Association between qualified ICFI and children’s physical development

In Table 2, the ANOVA results show a statistically significant difference in children’s length, weight, HAZ and WAZ among different ICFI groups (qualified ICFI groups and unqualified ICFI groups) (P<0.001, P<0.001, P<0.001, P<0.001). However, there was no difference in children’s WHZ among different ICFI groups (P = 0.541). Furthermore, the mean value for children’s length was 80.43 (7.66) cm for qualified ICFI groups and 77.94 (8.15) cm for unqualified ICFI groups (P<0.001). The mean value for children’s weight was 10.51 (1.94) kg for qualified ICFI groups and 9.98 (2.04) kg for unqualified ICFI groups (P<0.001). The mean value for children’s HAZ was -0.82 (1.40) for qualified ICFI groups and -0.92 (1.47) for unqualified ICFI groups (P<0.001). The mean value for children’s WAZ was -0.46 (1.11) for qualified ICFI groups and -0.54 (1.22) for unqualified ICFI groups (P<0.001).

**Fig 3:**
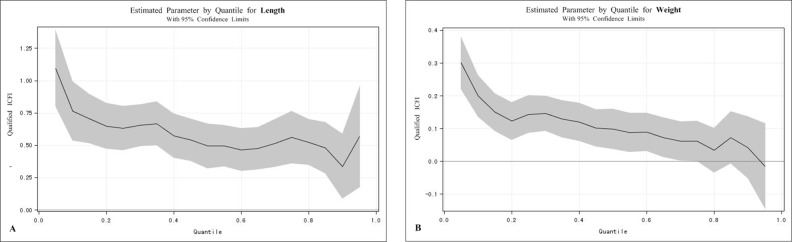
(A) This figure clearly indicates that regression coefficients varied by quantiles of children’s height after controlling for confounding factors. (B) This figure clearly indicates that regression coefficients varied by quantiles of children’s weight after controlling for confounding factors.

Table 3 shows the OLS and QR results obtained for children’s length, weight, HAZ, WAZ, and WHZ as outcomes variables. After adjusting for related variables, the β-estimates indicated that children’s length, weight, HAZ and WAZ were associated with qualified ICFI. The OLS model showed that qualified ICFI led to an increase in children’s length by 0.62 cm more than unqualified ICFI in all of the children. The QR model showed that children’s length was significantly associated with qualified ICFI from the 10th quantile to the 90th quantile. Moreover, the effect of qualified ICFI decreased as the child’s length distribution shifted from 10th quantile to 90th quantile (Fig 3). For example, for those in the three locations (10th quantile, 50th quantile and 90th quantile), qualified ICFI led to increases in children’s length of 0.76, 0.49 and 0.34 cm. In practical terms, the QR results suggested that qualified ICFI had a greater effect on children with unhealthy length, that is, greater than or equal to the 10th quantile (β = 0.76 cm).

**Table 3:** Association between qualified ICFI and children’s nutritional status using QR and OLS models for different quantiles of physical development.

		Length	Weight	HAZ	WAZ	WHZ
poor	10th	**0.76 (0.53 to 0.99)**	**0.20 (0.14 to 0.26)**	**0.17 (0.10 to 0.24)**	**0.14 (0.08 to 0.21)**	0.05 (-0.02 to 0.13)
	20th	**0.65 (0.47 to 0.83)**	**0.12 (0.07 to 0.18)**	**0.09 (0.03 to 0.15)**	**0.10 (0.04 to 0.15)**	0.03 (-0.03 to 0.10)
	30th	**0.65 (0.49 to 0.82)**	**0.15 (0.09 to 0.20)**	**0.09 (0.04 to 0.15)**	**0.08 (0.02 to 0.13)**	0.00 (-0.06 to 0.05)
intermediate	40th	**0.58 (0.40 to 0.75)**	**0.12 (0.06 to 0.18)**	**0.07 (0.02 to 0.12)**	**0.05 (0.01 to 0.10)**	-0.04 (-0.10 to 0.02)
	50th	**0.49 (0.32 to 0.67)**	**0.10 (0.04 to 0.16)**	**0.06 (0.01 to 0.11)**	0.03 (-0.01 to 0.08)	-0.05 (-0.10 to 0.00)
	60th	**0.47 (0.30 to 0.63)**	**0.09 (0.03 to 0.15)**	**0.07 (0.02 to 0.12)**	0.03 (-0.02 to 0.08)	-0.05 (-0.10 to 0.00)
good	70th	**0.52 (0.33 to 0.70)**	**0.06 (0.00 to 0.12)**	**0.10 (0.04 to 0.16)**	0.02 (-0.03 to 0.06)	-0.05 (-0.10 to 0.00)
	80th	**0.52 (0.35 to 0.70)**	0.03 (-0.04 to 0.10)	**0.10 (0.03 to 0.16)**	0.01 (-0.04 to 0.06)	-0.05 (-0.11 to 0.02)
	90th	**0.34 (0.09 to 0.59)**	0.04 (-0.05 to 0.14)	**0.11 (0.04 to 0.19)**	-0.01 (-0.08 to 0.06)	-0.03 (-0.11 to 0.05)
OLS		**0.62 (0.46 to 0.77)**	**0.11 (0.06 to 0.16)**	**0.11 (0.06 to 0.17)**	**0.07 (0.03 to 0.11)**	-0.01 (-0.05 to 0.04)

CI: confidence interval; OLS: ordinary least squares; QR: quantiles regression; values are β-estimates (95%CI);

Ethnic group, province, county rank, wealth index, mother’s education, mother’s age, parity, child's gender and child's age were adjusted in QR and OLS models.

The OLS model showed that qualified ICFI led to an increase in children’s weight by 0.11 kg more than unqualified ICFI in all of the children. The QR model showed that children’s weight was significantly associated with qualified ICFI from the 10th quantile to the 70th quantile. Moreover, the effect of qualified ICFI decreased as the child’s weight distribution shifted from the 10th quantile to the 70th quantile (Fig 3). For example, for those in the three locations (10th quantile, 40th quantile and 70th quantile), ICFI led to increases in children’s weight by 0.20 kg, 0.12 kg and 0.06 kg. In practical terms, the QR results suggested that qualified ICFI had a greater effect on children with unhealthy weight, that is, greater than or equal to the 10th quantile (β = 0.20 kg). This trend in children’s weight was consistent with the trend in children’s length.

Children’s HAZ was significantly associated with qualified ICFI from the 10th quantile to the 90th quantile. Moreover, the effect of qualified ICFI decreased as the child’s length distribution shifted from the 10th quantile (β = 0.17) to the 20th quantile (β = 0.09), and the effect of qualified ICFI remained consistent from the 20th quantile (β = 0.09) to the 90th quantile (β = 0.11). In practical terms, the QR results suggested that qualified ICFI had a greater effect on children with unhealthy HAZ, that is, greater than or equal to the 10th quantile (β = 0.17).

Children’s WAZ was significantly associated with qualified ICFI from the 10th quantile to the 40th quantile. Moreover, the effect of qualified ICFI decreased as the child’s WAZ distribution shifted from the 10th quantile (β = 0.14) to the 90th quantile (β = -0.01). In practical terms, the QR results suggested that qualified ICFI had a greater effect on children with unhealthy WAZ, that is, greater than or equal to the 10th quantile (β = 0.14). However, a significant association between qualified ICFI and children’s WAZ from the 50th quantile to the 90th quantile was not observed. However, the effect of qualified ICFI decreased as the children’s WHZ distribution shifted from the 10th quantile (β = 0.05) to the 90th quantile (β = -0.01).

From these results, we concluded that qualified ICFI was positively associated with children’s physical development in length, weight, HAZ and WAZ. As physical status worsened, the effect of qualified ICFI on children’s length, weight, HAZ, WAZ and WHZ increased.

## Discussion

### Summary of main results

Our findings indicated that qualified ICFI was positively associated with children’s physical development in length, weight, HAZ and WAZ in rural western China. Qualified ICFI was associated with lower risk of child malnutrition. As physical status worsened, the effects of qualified ICFI on children’s length, weight, HAZ and WAZ increased. These findings were important in that infants and young children, and their ICFIs can be used to identify children at lower risk for malnutrition.

### Association between ICFI and children’s physical development

Currently, most research has focused on understanding the relationship between ICFI and Z values (HAZ, WAZ and WHZ) and thus has indirectly aimed to understand the relationship between ICFI and nutritional status in infants and young children. Our results demonstrated that ICFI was significantly associated with height, weight, HAZ and WAZ. Children of the qualified ICFI had higher height, weight, HAZ and WAZ than children of the unqualified ICFI (β = 0.62 cm; β = 0.11 kg; β = 0.11; β = 0.07). The ICFI was significantly associated with weight and HAZ especially from the 10th quantile to the 90th quantile. Other reports have suggested that the ICFI was significantly associated with HAZ and WAZ in several countries including India, Burkina Faso and parts of Latin America [15–17]. In India, Neha Lohia and Shobha A Udipi constructed an ICFI scoring system as described by Ruel and Moursi et al. They found that the ICFI was significantly associated with the length-for-age z scores (LAZ) of children aged 6–24 months [15]. In Burkina Faso, Sawadago et al. also found that the ICFI was significantly and positively related to HAZ in children aged 6–23 months [17]. In China, related research was conducted by Ma et al. Their study found that the ICFI was positively associated with LAZ and WAZ among 18-month-old children in urban Shanghai [18].

In our study, we found that the quality of complementary feeding played a substantial role in poor physical development. In percentiles of poor physical development, the coefficients between the ICFI and complementary feeding indicators were larger than in percentiles of good physical development. The effect of qualified ICFI decreased as children’s HAZ distribution shifted from the 10th quantile (β = 0.17) to the 90th quantile (β = 0.11).

Additionally, the ICFI was significantly associated with WAZ from the 10th quantile (β = 0.14) to the 40th quantile (β = 0.05). In GEE models, children with qualified ICFI were less likely to be classified as stunting, underweight, wasting and overall malnutrition than children with unqualified ICFI (OR = 0.77, 95% CI = 0.69–0.85; OR = 0.75, 95% CI = 0.64–0.86; OR = 0.81, 95% CI = 0.67–0.98; OR = 0.76, 95% CI = 0.69–0.84). For poor physical development of infants and young children, feeding quality was more important, and physical development was more likely to be influenced by feeding. Guidance on feeding practices should be produced by health professionals to reduce malnutrition in rural western China.

### Application of ICFI in evaluating feeding practices of infants and young children

Ruel [8] first proposed the concept of an Infant and Child Feeding Index (ICFI) and the development of a multi-dimensional assessment scale with five indicators, including breastfeeding, bottle feeding or none, type of food, frequency of feeding food, general feeding frequency. The ICFI was primarily used for infants and young children between 6–36 months old who were divided into 3 subgroups: 6–8 months, 9–11 months, and 12–35 months. The children’s growth was closely linked to complementary feeding practices at 6–36 months. Rapid growth in infants and young children at 6–36 months often led to malnutrition. Other scholars’ studies on the ICFI have expanded to examine variables such as time of complementary feeding and dietary diversity. Most formulas to calculate ICFI have been based on the WHO feeding guidelines, taking into account differences by region. Qualified ICFI was defined as a feeding index score that equated to a proportion greater than 60% of the total score.

The ICFI in this paper was based on the WHO feeding guidelines and took into account the ICFI formula developed by Ruel and Menon and the ICFI scoring system developed by Lai Jian-qiang for use in China. Breastfeeding, bottle feeding or none, type of food, frequency of feeding food, general feeding frequency, first complementary feeding time and dietary diversity were the main indicators in this study’s multi-dimensional assessment. In this study, the ICFI was divided into 3 age groups: 6–8 months, 9–11 months and 12–35 months. To take into account the recommendations of the National Institute for Nutrition and Health, Chinese Center for Disease Control and Prevention, qualified ICFI referred to a score exceeding 60% of the total ICFI score. In this paper, qualified ICFI > 13.8 and unqualified ICFI ≤ 13.8.

### Strengths and limitations

The major strength of the study was the use of QR. From the minimum to the maximum response, this regression model for the height, weight, HAZ, WAZ and WHZ of children offered a more comprehensive view than other regression models of the relationship between ICFI and physical development. Using multiple quantiles in the model, we were able to gain a more complete understanding of how the height, weight, HAZ, WAZ and WHZ distributions were affected by the ICFI. Further strengths of this study included the ICFI scoring system used in this paper, which was based on the following: the WHO feeding guidelines, the ICFI formula developed by Ruel and Menon and the ICFI scoring system developed by Lai Jian-qiang for use in China. Hence, the ICFI was able to properly assess children’s feeding practices in this study.

This study also had some limitations. Recall bias existed in this study because all of the feeding information was obtained through mothers’ recall. Additionally, as this was a cross-sectional study, the infants and young children were of different ages at the time of the survey, which might have affected the accuracy of estimating the magnitude of complementary feeding practices.

## Supporting information

S1 File Blank copy of the questionnaire (in English). (PDF)
