# Supplementary material for: Association between the Infant and Child Feeding Index (ICFI) and nutritional status of 6- to 35-month-old children in rural western China
Source: PLoS One. 2017 Feb 16;12(2):e0171984. doi: 10.1371/journal.pone.0171984 (PMC5313129; doi:10.1371/journal.pone.0171984)
Supplement: S1 File — (PDF) [file pone.0171984.s001.pdf]

Variables	Contents	Code
BD	Survey Date：2005 (year)______(month)_______(day)	2005/_ _/_ _
Demographic characteristics		
B1	Family size:______	__ __
B2	Parity:________	__
B3	Mother’s race:______	
B4	Mother’s birth date: _ _ _ _/_ _/_ _	_ _ _ _/_ _/_ _
B5	Mother’s educational period:______year	__ __
B6	Father’s educational period:______year	__ __
B7	Water source:______	
B8	Household Income:______	
B9	Household Television Sets: (1) none (2) black and white (3) color	__
B10	Household Vehicles: (1) none (2) bicycle (3) motorcycle (4) tractor or car (5) others_____	__
Feeding		
B11	Child birth date: _ _ _ _/_ _/_ _	_ _ _ _/_ _/_ _
B12	Child sex: (1) boy (2) girl	__
B13	Breastfeeding: (1) yes (2) no	__
B14	When was your child weaned:__________month	__ __
B15	When did the child begin to drink water:_______month	__ __
B16a<br><br>B16b	When did the child begin to add grains:_______month<br><br>If adding，adding frequency：<br>(1) ≤1 time/month	— —<br><br>__

	(2) 1 time/month<br>(3) 2-3 times/month<br>(4) 1 time/week<br>(5) 2-3 times/week<br>(6) Every day	
B17a<br><br>B17b	When did the child begin to add egg:_______month<br><br>If adding，adding frequency：<br>(1) ≤1 time/month<br>(2) 1 time/month<br>(3) 2-3 times/month<br>(4) 1 time/week<br>(5) 2-3 times/week<br>(6) Every day	— —<br><br>__
B18a<br><br><br>B18b	When did the child begin to add fresh milk:<br>_______month<br><br>If adding，adding frequency：<br>(1) ≤1 time/month<br>(2) 1 time/month<br>(3) 2-3 times/month<br>(4) 1 time/week<br>(5) 2-3 times/week<br>(6) Every day	— —<br><br>__
B19a<br><br><br>B19b	When did the child begin to add fresh milk powder:<br>_______month<br><br>If adding，adding frequency：<br>(1) ≤1 time/month<br>(2) 1 time/month<br>(3) 2-3 times/month<br>(4) 1 time/week<br>(5) 2-3 times/week<br>(6) Every day	— —<br><br>__
B20a	When did the child begin to add formula:<br>_______month	— —<br><br>__

B20b	If adding，adding frequency：<br>(1) ≤1 time/month<br>(2) 1 time/month<br>(3) 2-3 times/month<br>(4) 1 time/week<br>(5) 2-3 times/week<br>(6) Every day	
B21a<br><br><br>B21b	When did the child begin to add bean products: _______month<br><br>If adding，adding frequency：<br>(1) ≤1 time/month<br>(2) 1 time/month<br>(3) 2-3 times/month<br>(4) 1 time/week<br>(5) 2-3 times/week<br>(6) Every day	— —<br><br>—
B22a<br><br><br>B22b	When did the child begin to add fish:_______month<br><br>If adding，adding frequency：<br>(1) ≤1 time/month<br>(2) 1 time/month<br>(3) 2-3 times/month<br>(4) 1 time/week<br>(5) 2-3 times/week<br>(6) Every day	— —<br><br>—
B23a<br><br><br>B23b	When did the child begin to add meat (beef, mutton and pork):_______month<br><br>If adding，adding frequency：<br>(1) ≤1 time/month<br>(2) 1 time/month<br>(3) 2-3 times/month<br>(4) 1 time/week<br>(5) 2-3 times/week<br>(6) Every day	— —<br><br>—
B24a	When did the child begin to add fruits and vegetables:	

B24b	________month<br><br>If adding，adding frequency：<br>(1) ≤1 time/month<br>(2) 1 time/month<br>(3) 2-3 times/month<br>(4) 1 time/week<br>(5) 2-3 times/week<br>(6) Every day	__ __<br><br>__
Anthropometric data		
B25	Mother's height__ __ __ .__cm	__ __ __ .__
B26	Total weight of mother and chid__ __ .__kg	__ __ .__
B27	Mother's weight__ __ .__kg	__ __ .__
B28	Child' weight__ __ .__kg	__ __ .__
B29	Child's length__ __ __.__cm	__ __ __ .__
